# The effects of acute coordinative vs. endurance exercise on the testosterone concentration

**DOI:** 10.3389/fphys.2026.1782332

**Published:** 2026-03-18

**Authors:** Henning Budde, Anett Mueller-Alcazar, Christiane Ahrens, Bruna Velasques, Pedro Ribeiro, Sergio Machado, Flavia Paes, Mirko Wegner

**Affiliations:** 1 Institute for Systems Medicine (ISM), MSH Medical School Hamburg University of Applied Sciences and Medical University Hamburg, Hamburg, Germany; 2 Institute for Cognitive and Affective Neuroscience (ICAN), MSH Medical School Hamburg University of Applied Sciences and Medical University Hamburg, Hamburg, Germany; 3 Bioscience Department, School of Physical Education of the Federal University of Rio de Janeiro, Rio de Janeiro, Brazil; 4 Institute of Applied Neuroscience, Rio de Janeiro, Brazil; 5 Brain Mapping and Sensory Motor Integration, Institute of Psychiatry, Rio de Janeiro, Brazil; 6 Panic and Respiration, Institute of Psychiatry, Federal University of Rio de Janeiro, Brazil and Laboratory of Physical Activity Neuroscience, Neurodiversity Institute, Rio de Janeiro, Brazil; 7 Department of Health Science, Institute of Human Movement Science, University of Hamburg, Hamburg, Germany

**Keywords:** acute exercise, coordinative exercise, endurance exercise, heart rate (HR), hypothalamic-pituitary-gonadal axis (HPG-axis), physical activity (PA), physical stress, testosterone

## Abstract

**Introduction:**

Physical exercise interventions are associated with neuroendocrine activation and transient changes in salivary testosterone (T) concentrations. Until now, most studies have focused on endurance exercise, but not on coordinative exercise (CE). The aim of this study is to examine the effects of two different interventions with an intraindividual comparison. We hypothesize that T concentration after an acute CE would be higher as an acute endurance exercise of the same intensity and duration.

**Materials and Methods:**

61 students between 18 and 30 years of age (*M* = 21.9, *SD* = 3.2) first completed a coordinative exercise and 7 days later an endurance exercise of the same intensity and length which was self-set on the first day, with a maximum heart rate of 64%–76% (HR_max_) over a period of 15 min. In order to measure changes in the hypothalamic-pituitary-gonadal axis activity, saliva samples were collected at baseline (t_1_), and then 5 and 30 min after the exercise (t_2_ and t_3_).

**Results:**

T levels changed significantly over time, *F*(2,106) = 10.418, *p* < 0.001, *eta* ^*2*^ = 0.164. T levels increased shortly after the exercise (t_2_) and decreased again at t_3_. There was no difference in T levels regarding the two exercise types, *F*(2,106) = 0.471, *p* = 0.496, *eta* ^*2*^ = 0.009.

**Conclusion:**

T levels increased in both conditions, shortly after exercise. The coordinative exercise do not result in a different T release compared to a endurance exercise of the same intensity.

## 1 Introduction

Acute physical exercise (PE) is associated with neuroendocrine responses and has been linked to increases in testosterone (T) concentration if the intensity of the intervention exceeds a certain threshold (Gronwald et al., 2018; Budde et al., 2010). Different modes of PE have gained increased attention in research over recent years (Knatauskaite et al., 2022; Leal et al., 2021a; Tu et al., 2026). PE refers to targeted, planned, and structured forms of physical activity (PA) (Amatriain-Fernandez et al., 2020). PE should be differentiated between acute PE (single bout) and chronic PE (exercise training) (Budde et al., 2025a).

In the event of acute physical or psychological stress, our body’s stress response essentially occurs *via* the hypothalamic-pituitary-gonadal axis (HPG-axis), which can primarily be characterized by the release of T (Budde et al., 2010). T has multiple effects on metabolism and is known as an anabolic hormone (Budde et al., 2010). The circadian rhythm of T is stable and is only slowly influenced by external conditions (Fenn et al., 2026; Tu et al., 2026; Zuloaga et al., 2024). During acute physical stress, a peak secretion of T can be observed approximately 20 min after the onset of the stimulus, whereas no significant changes were observed at lower exercise intensities (D'Andrea et al., 2020). If the organism is unable to terminate the stress response, e.g., in the case of chronic stress exposure, stress-adaptive mechanisms can lead to pathological changes (Leal et al., 2021b; Radley and Herman, 2023; Wegner et al., 2014).

Several training studies have shown that chronic endurance training has been associated with long-term differences in T concentration and has been discussed in relation to preventive and therapeutic outcomes in reducing the experience of stress (D'Andrea et al., 2020). This shows that high levels of PA (which is measured in this study, too) are related to a lower physiological stress reactivity. The acute and chronic neuroendocrine stress responses induced by PE are not yet fully understood. Results of studies comparing different types of chronic interventions have shown that coordinative exercise (CE) were leading to a similar cortisol awakening response (Wegner et al., 2019).

Based on current research, the question of whether there are direct effects of different acute interventions of the same intensity (i.e., with the same cardiovascular load) on the T concentration will therefore be investigated. The intensity of the acute activity is considered the primary physiological factor for determining the neuroendocrine response (Gronwald et al., 2018). In connection with psychosocial stress, the T response appears to be even more distinctive (Wegner et al., 2014). A distinction is made between acute physical exercise of a coordinative nature and acute physical endurance exercise. It is assumed that stress load will be higher during the CE intervention. This is, for example, shown in a similar study but with the assessment of C. The effect C has on the HPG axis and on testosterone secretion, can also be demonstrated for T on the hypothalamic–pituitary–adrenal (HPA) axis and on its cortisol secretion (Zueger et al., 2023). In addition to the intensity of the stressors, the type of stressor itself also has an influence on T levels and that the additional psychosocial stress can increase T levels significantly more than purely physical stress of moderate intensity (65%–75% HRmax) (Domes et al., 2024; Tu et al., 2026). To our knowledge, there have been no studies on whether T concentration differs between an acute CE and an acute endurance exercise of the same intensity and duration.

The reaction to physical interventions is demonstrably individual and depends on many influencing factors (Gronwald et al., 2019). For this reason, the intra- and inter-individual variability should be given special consideration here (Herold et al., 2021) and influencing factors such as gender, PA and will be investigated exploratively.

We chose two stressors that were assumed to moderately but significantly change testosterone levels (i.e., endurance exercise at 64%–76% of the maximum heart rate, HRmax; and coordinative exercise of the same intensity). To implement the intervention in an educational setting we chose a duration of 15 min, which has previously been shown to affect steroid hormones in school children and adolescents (Wegner et al., 2014). We hypothesize that T concentrations following acute CE would differ from those observed after acute endurance exercise of the same intensity and duration.

## 2 Methods

The sample and intervention protocol described here are identical to those reported in Budde et al. (2025b), where cortisol responses were analyzed. The current study presents new and unpublished analyses focusing on testosterone concentrations. No testosterone data have been reported previously. Analyzing cortisol and testosterone separately is essential due to their opposing roles in the body’s metabolic balance.

### 2.1 Participants

A power calculation using G*Power (α = 0.05, 1 − β = 0.95, η^2^ = 0.08) indicated that 60 participants were required to detect a medium effect size in an ANCOVA with two factors and three repeated measures (Wegner et al., 2014; Windisch et al., 2012). Ethical approval was obtained prior to data collection from the Ethics Committee of MSH Medical School Hamburg (approval number MSH-2021/131, 28 September 2021). Furthermore, the study was preregistered. All participants provided written informed consent, and the study complied with the principles of the Declaration of Helsinki.

In total, 61 individuals aged 18–30 years participated in the study (32 female: M = 21.5, SD = 2.9; 29 male: M = 22.5, SD = 3.5; overall M = 21.9, SD = 3.2). Recruitment took place between September 1 and 31 October 2023, *via* university mailing lists, campus flyers, and word of mouth. Eligibility criteria included being in good general health and capable of performing moderate physical activity. Individuals with endocrine or metabolic disorders (polycystic ovary syndrome, congenital adrenal hyperplasia, diabetes, Cushing’s syndrome, thyroid disorders), obesity, ovarian cancer, or current hormone therapy were excluded.

Female participants were asked about their menstrual cycle phase and contraceptive use. As no significant effects of the menstrual cycle on testosterone responses were found, data from male and female participants were analyzed together. Moreover, since the present study employs a within-subject design, the focus is on individual variation of T levels in response to two modes of exercise.

### 2.2 Study design

#### 2.2.1 Rationale for non-randomized order

The study followed a quasi-experimental pre–post design with repeated measurements. Each participant completed both an endurance and a coordinative exercise session, allowing the examination of individual testosterone responses across the two conditions. To the best of our knowledge, a within-subject comparison between two modes of exercise has not been tested before. Randomization of session order was not possible, as the coordinative exercise (CE) needed to be conducted first to standardize exercise intensity. HR-matching required CE to be performed first to calibrate the endurance exercise (EE) intensity. This was done to control for the effect of exercise intensity and being able to focus on the effect of the two exercise modes. A randomization would have introduced errors in the EE intensity condition, reducing experimental control of intensity.

Exercise intensity was defined according to the classification of Garber et al. (2011) targeting the moderate range between 64% and 76% of maximum heart rate (HR_max_). HR_max_ was estimated using Tanaka’s formula (HR_max_ = 208–0.7 × age). The Borg Rating of Perceived Exertion (RPE) scale was used to assess subjective exertion. Heart rate was continuously monitored using a Polar H10 chest strap and Polar M430 watch (Kempele, Finland).

Participants were asked to refrain from physical activity for 24 h and from eating for 2 hours before testing. All sessions were conducted between 2:00 p.m. and 4:00 p.m., as previous research has shown no significant diurnal effects on testosterone during this period. Before each session, participants completed a demographic questionnaire and the Godin Leisure-Time Exercise Questionnaire (GLTEQ) to assess habitual physical activity.

#### 2.2.2 Coordinative exercise (CE)

The coordination exercise session lasted 15 min and was performed using a floor ladder raised 10 cm above the ground with ten squares measuring 45 × 45 cm. Participants carried out a series of step and jump sequences that increased in difficulty. The first round served as a familiarization phase, followed by progressively more complex movement patterns, including diagonal and single-leg jumps. After approximately 8 minutes, difficulty was gradually reduced. The session concluded with a balance task in which participants performed a single-leg stance (standing scale) for 20 s on each leg.

#### 2.2.3 Endurance exercise (EE)

After a 7-day interval, the endurance session took place at the same time and in the same indoor location, with group sizes of up to six participants. Exercise intensity was matched to each individual’s HR and RPE levels recorded during CE. After a 20-min acclimatization period and HR setup, participants ran continuously for 15 min on an indoor track while maintaining their target HR with feedback. RPE was recorded again using the Borg scale.

#### 2.2.4 Sample collection and biochemical analysis

Saliva samples were collected immediately before exercise (t1), 5 minutes after exercise (t2), and 30 minutes after exercise (t3). Samples collected using passive drooling using SaliCap tubes (IBL, Hamburg, Germany) and stored at −20 °C until analysis. FBiochemical analyses were conducted using immunoassay methods at the Technical University of Dresden. Intra-assay and inter-assay coefficients of variation were 1.0% and 5.3%, respectively.

#### 2.2.5 Data analysis

Two participants were excluded due to invalid testosterone samples and three for not achieving the required exercise intensity, resulting in a final sample of 56 participants (25 male, 31 female). Testosterone data were missing at the second time point for four participants. As this accounted for only 1.8% of all data, multiple imputation using Rubin’s method was used to replace missing values after confirming randomness (Little and Rubin, 2019).

To meet assumptions of normality and homogeneity, testosterone concentrations were log-transformed prior to analysis. A repeated-measures ANCOVA was performed with condition (CE vs. EE) and time (pre, post, 30 min post) as within-subject factors. Gender and BMI were included as covariates due to their potential influence on testosterone levels. Post hoc pairwise comparisons were adjusted using the Holm correction because of the limited number of *post hoc* comparisons and in order to avoid false positives for these health-related measures.

## 3 Results

### 3.1 Descriptives

Descriptive statistics for the data collected in this study can be found in Table 1. Age was positively correlated with PA and T levels in the sample. Men showed lower resting heart rates, were more physically active, and had higher baseline T levels than women.

**TABLE 1:** Means (M), standard deviations (SD), and intercorrelations between study variables; n = 56.

Variable	*M*	*SD*	2	3	4	5	6
1 Age	21.93	3.17	0.16	−0.06	−0.16	0.27*	0.31*
2 Gender	0.45	0.50		0.13	−0.28*	0.31*	−0.72**
3 BMI (kg/m^2^)	23.53	4.10			0.15	0.02	0.06
4 HR_rest_ (bpm)	72.32	10.08				−0.07	−0.22
5 GLTQ	43.63	24.28					0.17
6 T (t_1_, pg/mL)	25.52	24.61					

Gender (0 … female, 1 … male), BMI = body mass index in kg/m^2^, GLTQ , godin leisure time exercise questionnaire; HR = heart rate in bpm, t1 = before the intervention; T concentration is shown in pg/mL.

There was no difference in testosterone levels between first measurement points in the CE vs. endurance exercise condition, *t*(55) = −0.304, *p* = 0.762, *d* = −0.041.

### 3.2 Testosterone changes depending on type of exercise

**FIGURE 1:**
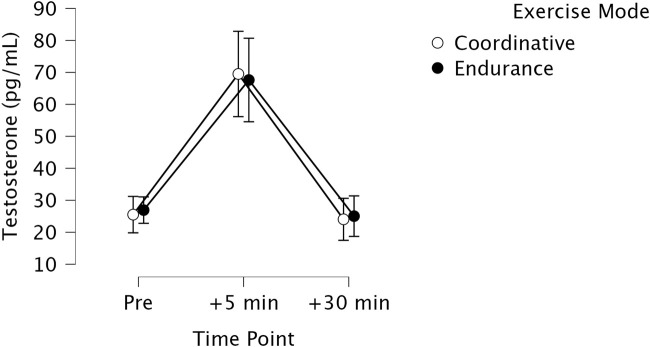
Changes in testosterone levels (T, in pg/mL) from pre exercise (pre) to after exercise (+5 min) and 30 min later (+30 min) for the two experimental conditions (1) endurance exercise (EE, black circles) vs. (2) coordinative exercise (CE, white circles).

**FIGURE 2:**
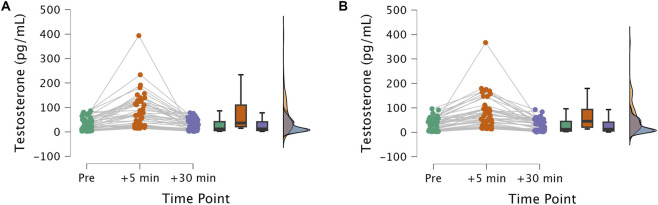
Raincloud plots for individual patterns of testosterone (T, in pg/mL) variation from pre exercise (pre, green) to after exercise (+5 min, orange) and 30 min later (+30 min, purple) for the two experimental conditions **(A)** endurance exercise (left) vs. **(B)** coordinative exercise (right).

T levels varied in response to the different types of exercise. Because of the gender differences in T, gender was included as a covariate in the analysis of co-variance. In a pre-analysis only BMI was shown to affect the pattern of results significantly and was therefore included as an additional covariate in the analysis. T levels changed significantly over time with a large effect size, *F*(2,106) = 10.418, *p* < 0.001, eta^2^ = 0.164. T levels increased shortly after the exercise and decreased again at t3 (see Figures 1,2). This pattern could be observed in both exercise conditions (CE and EE). There was no difference in T levels regarding the two exercise types, *F*(2,106) = 0.471, *p* = 0.496, eta^2^ = 0.009. The interaction effect between time x exercise type was moderate but not significant, *F*(2,106) = 3.033, *p* = 0.052, eta^2^ = 0.054. Regarding the covariates BMI and gender, it could be shown that the interaction effect of time x exercise type x BMI was significant, *F*(2,106) = 3.859, *p* = 0.024, eta^2^ = 0.068. Participants higher in BMI showed stronger T responses to endurance exercise compared to CE at t_2_. Participants lower in BMI showed stronger T responses to CE at t_2_. The interaction effect of time x exercise type x gender was non-significant, indicating that the pattern of change between exercise type over time was not gender specific, *F*(2,106) = 2.435, *p* = 0.093, eta^2^ = 0.044. Physical activity (GLTQ) did not significantly affect the results and was thus excluded from the final analysis.

Post-hoc tests with Holm correction for both coordinative and endurance exercise over the three time points show that there is no difference between testosterone values at t_1_ compared to t_3_, *t*s < 0.759, *p*s > 0.450, *d*s < 0.068. However, t_2_ significantly differed from t1, *t*s > 11.845, *p*s < 0.001, *d*s > 1.056, and from t3, *t*s > 12.604, *p*s < 0.001, *d*s > 1.123, for both exercise types.

Additional information on gender differences in testosterone levels can be found in Supplementary Table 1.

## 4 Discussion

Contrary to our hypothesis, CE did not result in a stronger testosterone response than endurance exercise. Although CE imposes higher demands, our results suggest that the cognitive-affective component of coordinative tasks was not sufficient to amplify HPG activation beyond the stimulus produced by moderate-intensity physical exertion alone. Recent models emphasize that testosterone reactivity depends primarily on exercise intensity, lactate accumulation, and metabolic stress (D'Andrea et al., 2020). These factors were intentionally matched between CE and endurance exercise, which may have minimized any potential additive effect of cognitive load. The endurance intervention was carried out at the same time of day 7 days after the coordinative exercise, between 64% and 76% HR_max_ (Garber et al., 2011) lasting 15 min.

We explain our acute results with the effect we observed when comparing different types of *chronic* interventions (T before and after a training 3 times a week over 10 weeks). This confirms the finding that coordinative training did not lead to a difference in T concentration compared to an endurance training (Akko et al., 2020). According to D’Andrea et al. (2020) regular acute exercise leads to adaptations that are reflected in a reduced physiological HPG response (D'Andrea et al., 2020). It would therefore be expected that enhanced T concentration acutely results in a reduced concentration after a chronic treatment.

Our findings align with contemporary models of HPA–HPG axis interplay, which emphasize that testosterone increases acutely in response to short-term metabolic and psychological stressors, but that the magnitude of this response is strongly moderated by the relative contribution of HPA-axis activation (cortisol) and sympathetic arousal. It is possible that different neurobiological signaling pathways are activated by different exercise interventions (Knatauskaite et al., 2022). Budde et al. (2008) observed significantly higher attention after 10 min of coordinatively demanding exercise compared to endurance exercise. CE was suggested to require perceptual and higher-level cognitive processes, such as attention, that are essential for mapping sensation to action and ensuring anticipatory and adaptive aspects of coordination (Voelcker-Rehage and Niemann, 2013). Although acute coordinative exercise has been associated with cognitive engagement in prior work (Budde et al., 2008), which can be reflected in endocrine responses (a higher amount of cortisol) (Budde et al., 2025b), but this is not observed by different concentrations of T after our two acute interventions.

After the interventions, T concentration was significantly increased in the second measurement (t_2_) after both conditions, which was performed 5 min after the end of the interventions. And then falls again back to pre-exercise conditions 30 min after PE (t_3_). This is true for both gender albeit at a descriptive level relatively less pronounced on female participants due to lower average T levels in women. This result is in line with those of comparable studies. After acute physical running exercise of a higher intensity (70%–85% HR_max_) over a period of 12 min in younger students, an increase in T concentration could be observed in between 5 min after the exercise (Budde et al., 2010). T is first secreted a few minutes after the onset of the physical stressor before it then rises continuously until the peak of secretion. This increase is detectable in both blood and salivary samples (Papacosta and Nassis, 2011). Meta-regression analysis showed that increase in T level after exercise was inversely associated with the time elapsed between the end of the exercise and hormone assessments. The main determinant of this increase is the intensity of exercise (D'Andrea et al., 2020). However, individuals respond differently to certain exercise stimuli (Gronwald et al., 2019; Knatauskaite et al., 2021).

In addition to the environmental influencing factors already listed could also be attributed to affect the T response. With regard to gender, there was a difference to be found in T secretion which is not surprising (Akko et al., 2020; Wegner et al., 2014). As expected, women showed lower average levels of T compared to men. However, gender did not affect the overall pattern of results showing an exercise related increase in T in both genders.

One limitation is that the present study did not assess cognitive outcomes. Therefore, any interpretation regarding cognitive or neuronal activation remains speculative. Further, due to our research question, we were unable to use a randomized controlled trial design. We first had to determine the individual HR during the CE to determine the target intensity of the second cardiovascular exercise in order to control exercise mode comparisons for intensity effects. Since with CE the possible maximum intensity is lower than with endurance exercise. It therefore needed to be determined first and properly underestimates the potential influence of a habituation effect. The lack of random assignment is the major limitation of the quasi-experimental study design (Iwahori et al., 2022). Another limitation was that we were not controlling the nutritional intake (Whittaker and Harris, 2022) and the resting time (Su et al., 2021) outside of the intervention which could have impacted the results. The participants were exercising in an interindividual comparable intensity according to Garber et al. (2011), we calculated their HR_max_ using a formula (Tanaka et al., 2001) and did not explicitly test their HR_max_, for example, with the shuttle run test. Another limitation of our study is the absence of a non-exercise control condition, which precludes isolating exercise-specific effects from potential time-dependent or methodological confounds common in salivary assays.

## 5 Conclusion

The results demonstrate that acute exercise in both endurance and coordinative mode activates the HPG axis, as shown by a temporary rise in T-levels. However, the higher demands of coordinative exercise did not change the hormonal response compared to endurance exercise. This suggests that short-term changes in T are more closely associated with physical exertion than with task complexity - an insight that can guide practitioners in designing flexible training interventions without compromising hormonal effects.

## Data Availability

The raw data supporting the conclusions of this article will be made available by the authors, without undue reservation.
