# Supplementary material for: The effects of acute coordinative vs. endurance exercise on the testosterone concentration
Source: Front Physiol. 2026 Mar 18;17:1782332. doi: 10.3389/fphys.2026.1782332 (PMC13038518; doi:10.3389/fphys.2026.1782332)
Supplement: Supplementary file 1 [file DataSheet1.pdf]

Supplementary Material

1 Supplementary Figures and Tables

1.1 Supplementary Figure

Exercise Mode	Time	Gen	N	Mean	SD
**Cardiovascular**	**t1**	Women	31	9.851	14.845
		Men	25	44.947	20.043
	**t2**	Women	31	30.561	29.98
		Men	25	117.772	75.498
	**t3**	Women	31	7.943	4.98
		Men	25	44.026	17.529
**Coordinative**	**t1**	Women	31	9.222	11.837
		Men	25	48.886	19.926
	**t2**	Women	31	41.053	67.045
		Men	25	100.546	42.968
	**t3**	Women	31	7.983	6.904
		Men	25	46.188	20.09

Supplementary Figure 1. Testosterone levels (M, SD; in pg/mL) of women vs. men for both exercise modes (cardiovascular vs. coordinative) before exercise (t1), five minutes after (t2), and thirty minutes after exercise (t3).
